# Exploring Attachment Differences Across the Contexts of Sports and Academics: A Qualitative Exploration of Child and Parent Experiences

**DOI:** 10.3390/bs14121153

**Published:** 2024-12-02

**Authors:** Sheng-I Chang, Ya-Hsin Lai

**Affiliations:** Master Program of Child and Youth Welfare, Chinese Culture University, Taipei 11114, Taiwan; b0703511@ulive.pccu.edu.tw

**Keywords:** attachment, contextual-specific differences, child–parent relationship, parenting, qualitative research, sport, academics

## Abstract

This study aimed to explore the nature of contextual differences in child–parent attachment relationships and examine how these experiences relate to children’s psychological outcomes. A theoretically informed qualitative study was conducted using semi-structured interviews with 15 participants across four groups of Taiwanese youths and parents, representing different contextual attachment combinations. Data were analyzed using a hybrid thematic analysis approach, integrating both inductive and deductive methods. The findings revealed two key characteristics of secure attachment across contexts: (1) parental timely and sensitive responsiveness enhances children’s openness to share personal thoughts, and (2) parental empathic and genuine concern fosters children’s empathy. Conversely, insecure attachment across contexts was marked by (1) inappropriate parental responses reducing children’s willingness to share their inner feelings, and (2) parental lack of empathy frustrating children and discouraging communication. In addition, parental beliefs about children’s achievement behaviors (e.g., expectation primarily centered on academic achievement and viewing athletic achievement as an alternative pathway to higher education) and parenting practices (e.g., performance- vs. mastery-oriented achievement goals, conditional negative regard, and psychological control through invalidating feelings and demeaning the child) significantly influenced children’s psychological outcomes, particularly in terms of basic psychological needs satisfaction and frustration (autonomy and competence) across academic and sports domains. These findings advance our theoretical understanding of contextual attachment dynamics and provide practical insights for fostering optimal parenting strategies, particularly in academic and sport-related contexts.

## 1. Introduction

The issue of individuals’ fluctuation in attachment schemata has recently been explored and understood from different perspectives (e.g., across developmental stages, across specific relationships) (e.g., [1,2,3,4,5,6,7,8,9,10,11,12]). Attachment-related experiences with primary caregivers (normally “parents”) at early developmental stages (i.e., from infancy until later adolescence) have been recognized to have considerable and prolonged influence on personal growth, interpersonal relationships, and psychological well-/ill-being [1,13,14]. However, scant research (e.g., [5,6,15]) has paid attention to the issue of “variation” in attachment security “within” specific child–parent relationships. Lai and Carr [5] have argued that even within specific relationships, a multilevel structure might be proposed that includes a generalized model of the given relationship, a model of the given relationship as it is experienced across different contexts, and a state-like fluctuation that functions episodically. These “context-specific” working models of attachment were conceptualized as schemata in which one’s attachment representations with parents could vary by context and therefore be stored and experienced contextually in a psychological and emotional sense.

Why should child–parent attachment representations vary across contexts? What kinds of contexts possess the potential to shape and sculpt child–parent attachment representations at the contextual level? Research suggests that the lives of many Western children often center around contexts such as academics and extracurricular activities, including sports, art, and music (e.g., [16,17,18,19]). Environmental characteristics, such as highly public and competitive settings, evaluation and reward systems, and interpersonal complexities, commonly emphasized in these contexts like “academics” and “sports”, can direct parental focus toward maladaptive goals and expectations for their children (e.g., [20,21]). This focus has been shown to impact children’s diverse psychological and behavioral costs (i.e., low psychological need satisfaction, need frustration, test anxiety, and competence-contingent self-worth; perfectionism; depressive symptoms; low task persistence; and performance-approach/avoidance goals and amotivation) (e.g., [22,23,24,25,26]). In the sports literature, for instance, parents who foster a performance-oriented climate—emphasizing ability, recognition, and superiority—are more likely to adopt controlling practices and unempathetic responses with their children (e.g., [16,27,28,29]). This motivational atmosphere often undermines children’s autonomy, competence, and relatedness, leading to negative emotions such as anxiety and stress when parental expectations are unmet (e.g., [30,31,32]). Such performance-focused cues may activate sport-specific child–parent attachment representations, which may not be as salient with the same parent in other contexts where secure attachment interactions exist. This suggests that distinct contextual cues can trigger specific attachment schemas within child–parent relationships.

Furthermore, a series of studies in the Western literature (e.g., [22,33,34,35,36,37,38]) have considered parental conditional regard (PCR) as context-specific socializing practices in achievement domains (i.e., academics and sports). Specifically, parental conditional positive regard (PCPR) occurs when parents are perceived to show increased affection, recognition, and attention when their child meets expected goals. Conversely, parental conditional negative regard (PCNR) is perceived when parents withhold affection, love, and respect if their child falls short of their expectations. PCPR and PCNR are recognized as disruptive parenting practices associated with maladaptive outcomes for children’s psychological functioning and relationships (e.g., anxiety and depression symptoms, contingent self-esteem, emotional dysregulation, resentment of parents, lower psychological need satisfaction, lower attachment security, and stable negative self-attributions) (e.g., [23,34,39,40,41,42,43]). It is plausible that domain-specific PCPR/PCNR may act as a contextual cue, primarily triggering insecure child–parent attachment schemas within that particular context.

Can children perceive differences in attachment interactions with their parents across various contexts? To date, it seems that only Lai and Carr [6,7] have sought to quantitatively examine variations in attachment security across the contexts of sports and academics in Taiwanese youths and to connect these global attachment patterns and psychological well-being indicators. Overall, their research found that (1) 80% of youth athletes (N = 385) can perceive variations in secure attachment-related characteristics across the contexts of sports and academics within a parent–child relationship, and (2) children’s perceived attachment quality in academics has a greater impact on context-specific and overall psychological indicators and parental attachment compared to the context of sports. It is interesting to note that Taiwanese youths’ perceptions of insecure attachment in academics appear to have a salient cross-contextual influence on their perceptions of sport-specific need frustration.

Although Lai and Carr’s [6,7] preliminary quantitative study provides initial evidence that contextual attachment operates within a hierarchical structure of parent–child relationships and significantly influences children’s context-specific and overall psychological outcomes, several areas warrant further exploration. First, while findings indicate that most youth participants can perceive notable differences in attachment security with their parents across sports and academic domains, simply averaging scores from a self-report contextual attachment scale (CAS; [6,7]) does little to illuminate the nature of these perceived differences. Specifically, what unique (or similar) attachment characteristics—whether psychological, cognitive, or behavioral—do children experience in these distinct contexts? To what extent are parents aware of any variation in attachment-related interactions with their children across these contexts? Furthermore, how can these differences be explained and understood? This study adopts a qualitative approach to delve into the limitations of prior quantitative analyses [6,7], thereby contributing to the research on the antecedents and outcomes of parent–child relationship quality in specific contexts (e.g., sports and academics), which is typically explored through a global attachment construct.

Moreover, this qualitative study examines school-aged youths in Taiwan, where cultural influences such as Confucian educational values, filial piety, and “face” culture differentiate Chinese culture from Western contexts. These influences shape parental beliefs and parenting norms—particularly in achievement-oriented areas—and also impact children’s responses and behaviors toward parental expectations (e.g., [44,45,46,47]). For example, previous research (e.g., [48,49,50,51,52]) on cultural differences in parental expectations for children’s academic achievement indicates that Chinese parents, including those in Taiwan, tend to place a stronger emphasis on academics than American parents. This emphasis manifests in setting high performance standards and dedicating significant time, effort, and resources to their children’s education. Confucian values, which prioritize education as a pathway to social status, heavily influence this focus. Additionally, Chinese youth are more likely than their American peers to accept parental guidance and strive to meet academic expectations as a way of honoring parents’ sacrifice and support, whether emotional or material, for their educational pursuits. Such culturally specific beliefs and parenting practices may lead to variations in child–parent attachment characteristics across achievement domains. A deeper understanding of attachment interactions within academic and sports contexts could support the development of culturally sensitive parenting strategies tailored to specific contexts rather than universal practices. Given the limited research addressing contextual attachment differences within specific parent–child relationships and the issues outlined above, this study aims to explore the following research questions: (1) What are Taiwanese children’s experiences of contextual attachment across the contexts of sports and academics? (2) What could explain these contextually different experiences in relation to children’s psychological outcomes?

## 2. Methods

### 2.1. Recruitment for Quantitative Investigation

#### Participants and Procedures

Two-step data collection for quantitative surveys and qualitative interviews was applied to this current study. In the first stage, a sample of 275 youth athletes was recruited from 17 schools and 21 different sports clubs in Taiwan. The criteria for recruitment included the following: (1) youths had been committed to attending training, practice sessions, and competitions for a given sport at least for one semester (normally 4–5 months) and (2) a chosen parent or primary caregiver was actively and substantially involved in his or her child’s sport- and academic-related activities for at least one semester. In total, 256 valid surveys (age range = 9–17 years; 62% boys, M_age =_ 13.74 ± 2.63) with signed consent were secured. Participants were recruited to achieve a balance between rural and urban areas and between seven major cities in Taiwan.

Youth participants represented their sports at four levels including club (14.5%), county/district (28.5%), national (53.1%), and international (3.9%) levels. In total, 26.6% of youth athletes had been involved in their current sport between six months and one year, and 73.4% of them participated in their sport for above one year. The youths reported spending a mean number of 10.48 h (SD = 6.03) in sport-related activities (e.g., training and competitions) per week during term time and 17.24 h (SD = 10.53) per week during off-term time. Around 10% of parents were involved in their children’s sport as a coach (8.6%) or parents had previously engaged in the same sport (as athletes) as their children’s current sport (11.7%). In total, 16.4% of youth athletes indicated that they had won personal prize money in their current sport.

Once permission for data collection from schools and signed consent from parents and young participants were obtained, surveys were conducted by the research team. Youths were asked to complete anonymous self-report measures in class or a quiet place in the school (without parents present). All participants were informed that they could refuse or withdraw their participation at any time. Ethical approval was obtained from the author’s institutional ethics committee.

### 2.2. Measures

#### 2.2.1. Contextual Child–Parent Attachment

The traditional Chinese version of the contextual attachment scales in sports (CAS-S) and contextual attachment scales in academics (CAS-A) [6] were applied to measure youth participants’ sport-specific and academic-specific attachment patterns with the selected parent. CAS-S consists of a 2-factor model with a set of 7 items, representing 3 items for *secure* style (e.g., “I feel secure and close to my parent in sport”) and 4 items for *insecure-avoidant* style (e.g., “I feel as though I can’t talk my sport-related feelings over with my parent”). CAS-A consists of a 2-factor model with a set of 8 items, representing 4 items for *insecure* style (“I often get into arguments with my parent when we talk about my schoolwork”) and 4 items for *secure* style (“I feel like my parent cares about my school life”). Youth participants were asked to rate each item on a 5-point Likert scale ranging from 1 (*strongly disagree*) to 5 (*strongly agree*). The total score of each of the dimensions in CAS-S and CAS-A was calculated by averaging the sum of the subscale items.

#### 2.2.2. Clustering Among Contextual Attachment Patterns

In order to explore the different combinations of attachment patterns that youths perceived for their nominated caregiver in the contexts of sports and academics, a two-stage method of cluster analyses (see [53]) was used to partition the sample into various clusters based on their scores for (a) CAS-S security and insecurity for their nominated caregiver and (b) CAS-A security and insecurity for their nominated caregiver. In the first stage, Ward’s hierarchical clustering method was conducted *twice* to obtain dendrograms and agglomeration schedules, resulting in a two-cluster solution in each of the separate contexts of sports and academics.

Next, two K-means cluster analyses were utilized to verify the initial 2-cluster seeds generated in the first stage. For the two-cluster analyses, if the values of the final cluster centers for a given variable were greater than the sample mean by 0.5 SD, then we labeled the cluster as “high” for that variable; if the values were less than the mean by 0.5 SD, then we labeled the cluster as “low” for that variable; and if the values were within a range of ±0.5 SD from the sample mean, then we labeled the cluster as “moderate” for that variable. Table 1 outlines the two-cluster solutions that we identified for each of the cluster analyses. For the CAS-S cluster analysis, cluster 1 reflected “*low* sport security and *high* sport insecurity” and cluster 2 reflected “*high* sport security and *low* sport insecurity”. For the CAS-A analysis, cluster 1 reflected “*low* academic security and *high* academic insecurity” and cluster 2 reflected “*high* academic security and *low* academic insecurity”.

Using these cluster analyses as a starting point, the whole sample was then explored in relation to the combination of youths’ attachment profiles for each of the contexts of sports and academics. For example, if a participant fell into cluster 1 for the sporting cluster analysis and cluster 2 for the academic cluster analysis, then they might be seen to reflect a profile of “low security in the sporting context and low security in the academic context”—which we operationalized as their within-parent attachment profile. Exploring the sample in this way, four different combination groups emerged: (1) high security (but low insecurity) in sports and academics—contextually consistent security, (2) high insecurity (but low security) in sports and academics—contextually consistent insecurity, (3) high security (low insecurity) in sports and low security (high insecurity) in academics—security in sports and insecurity in academics, and (4) low security (high insecurity) in sports and high security (low insecurity) in academics—insecurity in sports and security in academics. It is noteworthy that combinations (3) and (4) (n = 74 youth, 30% of sample) were suggestive of participants who experienced their nominated parent as significantly different in an attachment sense between the contexts. Table 2 displays the labels of these four combinations in tabular form.

### 2.3. Recruitment for Qualitative Investigation

#### Participants and Procedures

In the second stage of data collection, 54 youths were initially identified as a good fit for the follow-up interviews from the contextually different attachment combinations, which are profiles (3) and (4) in Table 2. After considering the balance of the children’s age and gender, as well as the possibility of being an information-rich source, eight young participants (age range = 10–17 years) and seven parents (one parent was the mother of two children categorized as having different attachment combinations) were selected as the suitable cases. All of the young participants were educated in mainstream schools (i.e., public/state schools), and half of them were from urban and the other half from rural areas in Taiwan. The youths had been trained in various sports clubs at school as national-level athletes. Two fathers who previously participated in the same sports as athletes as their children had engaged in their children’s sports as coaches in the past, but not within the most recent six months. A detailed description of each interviewee based on their demographic information is shown in Table 3.

In order to achieve information-rich conversations with the children and their assigned parents, pilot interviews were conducted with two mothers and one young participant separately by telephone. In addition to interview guides, youths’ responses to their sport/academic-specific attachment scales were also employed as a reference to facilitate them to reflect and compare their feelings and behaviors toward each context-specific relationship with their parent. Furthermore, those scales were also used as follow-up questions to clarify their responses to some interview questions which were not in accordance with the answers on the context-specific attachment scales.

All main interviews with youths and parents were individually conducted by telephone for three months. In total, fifteen in-depth semi-structured interviews (eight for youths and seven for parents) lasting an average of 40 min with audio taping were conducted individually at each of the participants’ preferred times as well as at a comfortable and quiet place. To avoid the social expectancy effect and provide a private and worry-free conversation, all of the young participants were interviewed first, followed by their assigned parents on the same or separate day. Parents were asked not to appear around while their children were doing interviews. The purpose of interviewing parents was to triangulate youths’ responses and also to enhance the richness and depth of this research. For examples of interview guides with children and their parents, refer to Table 4.

### 2.4. Analysis and Interpretation of Data

All of the interviews were conducted in Chinese and then sent back to each of the participants to initially check the accuracy. Subsequently, the data were interpreted by using both deductive and inductive thematic analyses guided by Boyatzis [54] and Braun and Clarke [55]. A deductive approach analysis was adopted to explore children’s attachment-related experiences across the contexts, and codes were identified by looking for evidence that appeared in line with previous theorists’ conceptualized attachment-related characteristics (e.g., [1,13,14,56,57,58,59]). Illustrative examples and clusters of codes in each of the separate contexts of sports and academics were identified to define themes and subthemes, respectively, from the dataset of all fifteen youth and parent interviews.

For the first research question, the themes and relevant extracts reflecting attachment-associated characteristics of *secure* attachment in *both* contexts were critically compared and contrasted in order to understand the salient or shared parenting merits of exploring the similarities and differences between children’s sports and academic life. Subsequently, these *secure* attachment characteristics in specific contexts were then contrasted with children’s perceptions in the other *insecure* contexts. By doing so, we might be able to detect what important parenting differences distinguished children’s experienced attachment relationships across contexts. For the second research question, a hybrid approach combining inductive and deductive methods was used to seek the possibilities resulting in children’s experienced attachment differences across the contexts and associative psychological outcomes (guided by SDT). This follow-up analysis explored meaningful themes that especially emerged from the data of eight interviews with youths and parents of contextually different attachment relationships (i.e., group 1 and group 2). The trustworthiness of this study was examined by utilizing parents’ data as triangulation to increase the validity by verifying the children’s responses.

## 3. Results

### 3.1. What Are Children’s Experiences of Contextual Attachment Across the Contexts of Sports and Academics?

#### 3.1.1. Parental Timely and Sensitive Responsiveness Enhances Children’s Openness to Share Personal Thoughts

The characteristics linked to secure child–parent attachment show psychological, cognitive, and behavioral similarities across sports and academic settings. Salient quotations can be seen in Table 5. Specially, in the sports context, children with secure attachment feel that their parents respond to their needs with genuine, timely, and appropriate support—whether emotional encouragement or practical assistance (Quotation 1, 4). This secure foundation enables children to openly share both the highs and lows of their sports experiences with their parents, much like they would with close friends, without fear of disappointing them or losing their support due to poor performance (Quotation 1, 2, 4). Children sense that, as long as they behave well, their parents will not intrusively involve themselves in their sport-related activities. This unconditional care is also evident in SS2′s father’s responses, which highlight his sensitive awareness of his child’s needs, respect for their choices and interests, and his timely, responsive support (Quotation 3, 5). In an academic context, securely attached children show similar attachment-related characteristics in their interactions with parents. For example, they feel comfortable sharing their experiences and feelings about school, perceiving a genuine concern from their parents for their learning. Daily interactions convey a sense of understanding, with parents providing timely and appropriate emotional support (Quotation 6, 7, 8, 9). This positive cycle of interaction not only fosters a sense of autonomy in children’s learning but also enhances their empathy toward the challenges their parents face (Quotation 9). As a result, it deepens the children’s feelings of closeness, independence, and competence in their relationship with their parents (Quotation 6, 7, 8).

#### 3.1.2. Parental Empathic and Genuine Concern Fosters Children’s Empathy

Common characteristics of secure parent–child attachment can be observed across different contexts, as highlighted in this theme. For instance, in the sports context, securely attached children feel that their parents genuinely listen to them, empathize with their needs and desires related to sports, and are willing to adjust their initial reluctance toward their involvement. This enables parents to offer timely emotional support and express care. In turn, this helps children empathize with their parents’ concerns and better understand their parents’ actions (Quotation 1, 2). Similarly, in academic contexts, securely attached children feel that their parents understand the challenges and feelings they experience in learning. Instead of intervening directly, parents provide appropriate practical advice and encouragement, allowing children to feel supported without pressure in their studies (Quotation 3, 4). Salient quotations can be referred to in Table 6.

#### 3.1.3. Inappropriate Parental Responses Reduce Children’s Willingness to Share Their Inner Feelings

Compared to the secure attachment characteristics shown in a sports context (Quotation 1, 2, 3, 4, 5), children with insecure attachment in academic settings may feel that their parents lack interest in engaging deeply or understanding their emotions when they share school-related experiences. Alternatively, parents may respond habitually with anger, control, or even abusive, unempathetic reactions, leaving children feeling emotionally unsupported and distant from their parents. This misaligned interaction can lead children to lower their expectations for parental support and gradually lose the desire to share personal or difficult experiences. Communication may then become limited to basic updates that parents need to know as children seek to avoid provoking negative emotional responses from their parents.

*I didn’t feel like to share my school life with mum, we’d normally have opportunity to chat during our dinner time at home…sometimes I would just tell her what teachers asked for parents to do or my exams results, she wasn’t really responsive to me…I didn’t feel like sharing more things with her…cos I didn’t expect she would respond to me anyway…*

*(II4, boy–mother bond)*

*Me and my mum, we didn’t talk much…she didn’t ask about my studies much or what’s going on in my school life…sometimes I would chat a bit about my school things with her…but she didn’t say much back…*

*(II3, boy–mother bond)*

*I hoped mum and me could have had more positive interactions… I didn’t tell her my feelings, cos she had let me feel it was hard to be close to her…I hoped she could have been nice to me in my studies, didn’t behave too bossy…*

*(II3, boy–mother bond)*

*If he didn’t do well in exams, I would be very angry…I would beat him…then he would run away…*

*(II3’s mother)*

*When we were chatting, he would only report good things, but not bad things…I felt there might be something that worried him, but he didn’t feel like to talk, might be his studies was not on track, didn’t do homework or didn’t know how to do it…he might worry I would shout at him if he told me something unhappy or bad things, so it’s better not to mention it…*

*(II3′s mother)*

In contrast to the secure attachment characteristics observed in academic contexts (Quotation 6, 7, 8, 9), insecurely attached children in sports contexts often feel anxious and tense about their parents’ presence at their sporting events. This anxiety may arise from them perceiving their parents’ consistently high expectations regarding athletic performance, which are often accompanied by a controlling or performance-oriented approach to goal achievement. Furthermore, parents’ lack of genuine understanding of their children’s needs and inability to provide unconditional love can lead to children feeling hesitant to share their inner feelings with parents.

*He kept asking me to beat that guy, to win the game…he thought my ability is better than him… but he didn’t really compare the skills of others…it was just his intuition…*

*(IS8, boy–father bond)*

*I think if you are doing a competition, then you gotta win…I told him…that game looked like it was your game…why you lose it? Then he said he was nervous because I was there…something like that…*

*(IS8′s father)*

*When he came back home, he didn’t really say how’s it going in his training…sometimes if we knew he was going to a game, we would ask about this…but he didn’t really say anything.*

*(IS8’s father)*

#### 3.1.4. Parental Lack of Empathy Frustrates Children and Discourages Communication

In contrast to the secure attachment characteristics shown in the sports context (Quotations 1, 2), children with insecure attachment in academic settings often experience recurrent conflict when discussing schoolwork with their parents. These conflicts typically arise when parents repeatedly reject or criticize their children’s attempts to express their thoughts and feelings, particularly in moments when children feel misunderstood. Parents, interpreting their children’s academic performance from a subjective standpoint, often overlook their children’s struggles and needs and fail to empathize with their emotions. This lack of understanding and empathy prevents parents from offering constructive feedback or appropriate support, which in turn frequently triggers frustration and anger in the children’s responses.

*Sometimes when talking about his studies, we would get into arguments…cos he wasn’t really interested in some courses, sometimes when we talked about it, he would get angry…like he really didn’t like composition writing cos he isn’t smart enough to compose an essay easily like others…he couldn’t…he needed lots of time to complete it…then I would blame him…it’s like he wasn’t really focused on it. He would say he just couldn’t figure it out …yeah…sometimes I would say to him to pay attention on it. Then he would get angry sometimes…would say that he really didn’t know how to write.*

*(II4’s mother)*

The extracts from the children’s perspectives also suggest that when they tried to share their thoughts and feelings with their parents, they often faced misunderstandings and negative reactions. After repeated experiences of having their concerns dismissed, they began to feel disappointed and frustrated. Over time, with diminished hope that their parents could genuinely understand or empathize, they ultimately chose to avoid discussing academic performance with their parents entirely.

*We always got into arguments because of my exam reports…like I did study hard but I didn’t get good marks…then she would say I didn’t really work hard at all…I tried to explain it to her but it didn’t work at all…I would be very unhappy about it…I hoped she could try to understand me first…let me explain it first…if she still felt I wasn’t really doing well…then it’s fine. But whether it’s my problems or not…she just kept shouting at me…*

*(SI5, boy–mother bond)*

*I don’t think she could understand my feelings. I did try to let her know my feelings but she seemed not able to get it…so, I don’t feel like to talk about my feelings to her now…I just try to comfort myself…I think we’re not on the same page…I tried before…but we had different opinions…she thought I didn’t do my job [studies] well but I thought I had done enough…so…I don’t talk to her about this anymore, I don’t know what else I can say…*

*(SI5, boy–mother bond)*

Furthermore, in contrast to the secure attachment characteristics observed in academic contexts (Quotations 3, 4), children with insecure attachment in sports settings often feel that their parents are unable to genuinely accept their viewpoints or empathize with their emotions. Instead, parents impose their own expectations and demands, causing children to feel resistant and resentful when discussing their sports participation.

*In the beginning my mom asked me to join this sport, but I didn’t really wanna join cos I wasn’t interested in it at all…*

*(II3, boy–mother bond)*

*I asked him to join in this sport team because he is too laidback…but I knew he didn’t wanna join at all. I could feel he wasn’t okay with it…he wasn’t really interested in it…he also told me he wanted to quit, he didn’t wanna take part anymore. Then I told him it’s impossible. I hope he can keep doing this sport cos he is too laidback…needs some training.*

*(II3′s mother)*

*I would chat a bit about his sport, like, did you run faster a bit? Did your speed increase? Something like that…sometimes we got into argument, he would say something like…why don’t you do it.*

*(II3′s mother)*

### 3.2. What Could Explain These Contextually Different Experiences in Relation to Children’s Psychological Outcomes?

To further explore the factors that shape cross-contextual differences in parent–child attachment (e.g., secure attachment in sports and insecure attachment in academics, insecure attachment in sports and secure attachment in academics) and how these differences impact children’s psychological well-being across settings, the findings can be organized into three main themes: parental beliefs about children’s achievement behaviors, parenting practices, and children’s psychological outcomes in different achievement domains.

#### 3.2.1. Parental Beliefs About Children’s Achievement Behaviors

##### Expectation Primarily Centered on Academic Achievement

Children with secure attachment in sports but insecure attachment in academics often feel that their parents’ high expectations in academics stem from their own past academic success, especially when they performed better than others. For instance, SI7 (boy–father bond) shared, “*I felt my dad had much higher expectations for my studies because I did much better than others when I was in primary school*.” Similarly, SI6′s mother noted, “*…because her exam marks were a lot better than her brother, I had much higher expectations for her… I would ask her to maintain a certain level…*” In contrast, these parents, while focused on academic success, tend to hold more balanced and realistic expectations for their children’s sports involvement. As SI7 described, “*I just needed to have fun and do my best in my games, that’s all… he didn’t ask for much…*”.

##### Considering Athletic Achievement as an Alternative Path for Higher Education

Children with insecure attachment in sports but secure attachment in academics often perceive that their parents understand their lack of interest in academics, and therefore, do not hold high expectations for their academic performance. For instance, IS8 (boy–father bond) noted, “*He knew I wasn’t good at studying, so he didn’t ask for anything at all… even when I failed my exams, he was still fine with that*.” IS8’s father added, “*I felt he wasn’t interested in studying; he thought sports were more engaging… he did poorly in every exam… I just told him to… pass the exams, that’s enough.*”. However, parents with this attachment combination often shift their expectations toward the child’s athletic performance, seeing it as a viable route to college admission. As IS8 shared, “*He knew I wasn’t doing well in my studies, so he told me to focus on sports. Low exam marks were fine as long as they weren’t too low—I could still get into a good university*.” IS8 further explained, “*It was my dad who suggested that if I’m not excelling academically, I could consider using sports to get into a university. Afterward, he expected me to perform well…*”.

#### 3.2.2. Parenting Practices Across Contexts

##### Performance- vs. Mastery-Oriented Achievement Goals

Parents who display attachment differences across various contexts often hold distinct beliefs and practices based on their expectations for their children’s educational pursuits. In contexts where children perceive an insecure attachment, they sense a strong parental focus on achieving high grades or outperforming others. For instance, SI7 (boy–father bond) noted, “*When my studies were declining, my dad still wanted me to maintain the level I had in primary school. He expected me to be one of the top in my class… he had very strict requirements*.” IS8 (boy–father bond) also shared, “*He kept telling me to beat that guy, to win the game… he thought my ability was better than his*.” IS8 further explained, “*I think if you’re competing, you have to win… I told him… that game looked like yours… why did you lose it?*” In contrast, parents with a performance-oriented mindset do not demonstrate this approach in contexts where children feel securely attached. Instead, they prioritize whether their children enjoy the learning process and are motivated to improve their own skills. As SI7 alluded, “*He thought that if I enjoyed my games and put in effort, then that was fine*.”.

##### Parental Conditional Negative Regard

In contexts of insecure attachment, some parents show attention and approval that seems conditional, often based on whether the child meets specific expectations. One father expressed, “*I used to go to watch his games, but not anymore… because… every time I went, he played poorly [laughs], so I stopped attending*” (IS8′s father). This conditional response leads the child to feel that parental support hinges on meeting certain standards, prompting a need to constantly prove their worth to gain approval. The child reflected, “*When he was complaining… it made me feel really bad, like I was no good… I’d feel very down…*”; “*It’s nice when he attends my games, but I don’t necessarily play better… I feel pressured… like I have to prove I can play well… but I just can’t*” (IS8, boy–father bond). In contrast, in contexts of secure attachment, children feel their parents’ love and acceptance as unconditional, allowing them to focus comfortably on their learning without anxiety over parental involvement or approval. As IS8 described, “*I felt very relaxed and comfortable while studying… I didn’t feel… that he didn’t check on my studies because he didn’t care… because if I asked him about something I didn’t know, he would help me*”.

##### Parental Psychological Control: Invalidating Feelings and Demean the Child

In insecure attachment contexts, children frequently feel that when their performance does not meet parental expectations, they face blame or criticism. As one child expressed, “*I felt pretty bad when I lost games… because he would complain about why I lost to that kind of player… I’m taller than him… I should be better*” (IS8, boy–father bond). Despite children’s attempts to share their reasons, parents often dismiss these explanations, leading to increased pressure to change the child’s thoughts or behavior, often accompanied by even harsher criticism. As SI5 (boy–mother bond) shared, “*I did try to tell her how I felt, but she just didn’t understand… it felt like we’re not on the same page… she thought I wasn’t working hard enough in my studies, but I felt I had done my best*”; “*I studied hard but didn’t get good marks… then she’d say I wasn’t really working hard… I tried explaining, but it didn’t matter… I wished she’d listen and let me explain first… but instead, she just kept yelling*”. Even when parents are aware of their children’s true thoughts, they often ignore them, focusing instead on pushing the child to conform to their own standards and expectations. As one mother described, “*He doesn’t enjoy writing compositions because it’s hard for him… he needs a lot of time to finish them… so I would blame him… it’s like he wasn’t trying. He’d say he just couldn’t figure it out… sometimes I’d tell him to just pay more attention*” (II4′s mother). In contrast, children in secure attachment relationships report feeling free from these controlling behaviors and the self-critical pressure to fulfill parental expectations. As one child noted, “*I don’t feel he puts pressure on my studies because he knows I’m not interested in schoolwork. My friends’ parents are always pushing them to do after-school courses, but my dad doesn’t ask me to do that*” (IS8, boy–father bond).

#### 3.2.3. Children’s Psychological Outcomes: Basic Psychological Needs Satisfaction and Frustration—Autonomy and Competence

Children with contextual attachment differences perceive distinct parental beliefs and disciplinary behaviors regarding their achievements across various contexts. These perceptions contribute to varying psychological outcomes in different achievement contexts. For instance, children experiencing insecure attachment often feel they lack control over their choices in academic or sports activities, with their efforts appearing more motivated by parental expectations than by personal interest. As SI7 (boy–father bond) alluded, “*He would force me to study those difficult subjects which I wasn’t interested in…like Maths… he always sat beside me when I was studying Maths… it was really a big pressure*”. SI6′s mother further shared, “*Sometimes if she didn’t do well on her exams, I would ask her to put in more effort on her studies*”. Many feel compelled to strive harder to meet parental standards, often as a way to acknowledge their parents’ investments in their lives. As IS8 (boy–father bond) described, “*I felt like my dad has spent a lot of money and time on my sport. I can’t waste his money… I gotta motivate myself to be better…can’t lose games…*”. When they fall short of parental expectations, their parents’ disappointment and criticism can amplify the children’s own feelings of inadequacy, which in turn affects future performance. For instance, SI6′s mother alluded, “*She seemed to have high expectations for herself, if her score wasn’t at the top, she would feel she did poorly*”. IS8 (boy–father bond) also shared, “*He made me feel like I was really bad, I would feel very down… so in the next game, I wouldn’t play well either…*”; “*It made me feel that I had to win to feel good… I should do better… but it wasn’t really what I wanted…*”. In contrast, within secure attachment contexts, parents fully support their children’s interests and needs, allowing them the freedom to choose activities that resonate with them. As SI7 (boy–father bond) described, “*I felt like I could be myself in sport all the time… didn’t really need to worry about anything or feel pressured…*”. This supportive environment enhances children’s confidence in their abilities, along with their actual performance outcomes. As IS8′s father alluded, “*He told me he couldn’t catch up in English, so I arranged some after-school courses for him on weekends… I followed up on his progress… he told me he was doing well…*”.

## 4. Discussion

The aim of this study was to enhance our understanding of the nature of contextual attachment within child–parent relationships through a qualitative exploration of two main questions—(1) What are children’s experiences of contextual attachment across the contexts of sports and academics? (2) What could explain these contextually different experiences in relation to children’s psychological outcomes?

Our findings suggest that when comparing characteristics of secure parent–child attachment interactions across different achievement contexts, children’s perceptions of their parents’ responses, parents’ views of the child, children’s self-evaluations, and responses to and expectations of parental behaviors appear to revolve around two primary themes: “*Timely and sensitive parental responsiveness enhances children’s openness to sharing personal thoughts*” and “*Genuine*, *empathic parental concern fosters children’s own empathy*”. Specifically, when facing difficulties or needs in sports or academics, children in secure attachment contexts are more open and willing to communicate, confidently expressing perspectives that may differ from those of their parents. This openness emerges from a sense that their parents are receptive, understanding of their needs, empathetic toward their frustrations in sports and academics (e.g., training setbacks and low interest in studies), and willing to provide constructive feedback that makes children feel valued. Conversely, when comparing secure attachment characteristics with those observed in insecure attachment contexts, we see that emotional, cognitive, and behavioral differences tend to cluster around two contrasting themes: “*Inappropriate parental responses reduce children’s willingness to share their inner feelings*” and “*Lack of parental empathy leads to frustration in children and discourages communication*”. For instance, children in insecure attachment settings often have low expectations of empathetic responses from their parents and are generally unwilling to share their feelings or express divergent views. This reluctance is linked to frequent experiences of parental responses that are emotionally reactive, dismissive, or overly controlling in sports and academic contexts rather than attuned and supportive.

These attachment-related patterns are consistent with theoretical perspectives (e.g., [1,13,14,56,57,58,59]) that describe secure and insecure child–parent attachment characteristics. Ainsworth [57], for instance, proposed that children with secure attachment to their primary caregiver develop positive working models for seeking closeness and security, grounded in consistently attentive, empathetic, and supportive responses to their emotional needs, particularly during times of vulnerability. Such secure parental responses enable children to view themselves as worthy of love and support and to confidently seek emotional comfort from their parents in times of stress. Furthermore, when compared with previous scales assessing attachment relationships in specific contexts—such as sports, academics, and physical activities—using either global (e.g., [60,61,62,63,64,65,66,67,68]) or context-specific (e.g., [6,7]) attachment measurements, the constructs and items describing attachment characteristics did not differ from those identified in this study. Our findings support the validity of Lai and Carr’s [6,7] traditional Chinese contextual attachment scales (i.e., CAS-S and CAS-A), suggesting that the fundamental dynamics of child–parent attachment—whether examined at a global level or within specific contexts—are consistently reflected in the cycle of children’s perceptions of parental responses, self-evaluations, and responses to and expectations of parental behaviors, all of which underscore common attachment characteristics.

When further exploring how children with contextually different attachment patterns (i.e., secure in sports and insecure in academics and insecure in sports and secure in academics) experience parent–child interactions across different settings, it becomes clear that these experiences are shaped largely by parents’ beliefs about their child’s achievement behaviors in each context. Specifically, regardless of the type of contextual attachment difference, parents often first assess the likelihood that their child can use academic performance as a primary pathway to further education—that is, whether the child’s academic achievements are sufficient for admission to a decent school. This assessment is typically based on the child’s past academic outcomes. If a child has previously performed well academically, parents may perceive them as having potential in academics, which leads to high parental expectations for the child to sustain a strong academic performance without necessarily expecting notable accomplishments in sports. On the other hand, if parents feel that the child’s past academic performance has been weak, they may judge the chances of getting into a desirable school through regular academic exams to be low. In this case, they might shift their focus, expecting that the child could gain admission to a reputable school through sports achievements instead. Interestingly, this expectation of sports success as an alternative path is often not due to the child’s strong past performance in sports, but rather because the child’s academic achievements are viewed as insufficient for successful school admission. Consequently, parents in these cases may set more realistic expectations for academic performance.

Our findings underscore the emphasis that Taiwanese parents place on academic achievement as a core educational value, as well as a primary path to upward mobility. It also illustrates how parents’ beliefs about their child’s academic and sports participation significantly influence the child’s own beliefs about their achievement behaviors. Cross-cultural studies examining the impact of parenting beliefs and behaviors on children’s academic values and achievements suggest that Taiwanese parents’ educational expectations are strongly influenced by Confucian beliefs, which emphasize that education is essential for achieving higher social status (e.g., [50,51,52]). As a result, Taiwanese parents often set high academic standards for their children. Taiwanese youths, growing up with these values, appear to internalize the importance of striving for academic excellence. Whether in academics or sports, they aspire to gain admission to prestigious schools through exceptional performance.

Parents with contextually different attachment relationships with their children appear to adjust their parenting practices based on their expectations for their children’s achievement behaviors in various contexts, aiming to ensure their future admission to prestigious schools. Our study found that in contexts where children perceive an insecure attachment, they often sense a strong parental emphasis on achieving high grades or outperforming others as measures of success. This emphasis may stem from environmental characteristics, such as highly public and competitive settings, evaluation and reward systems, and complex interpersonal dynamics present in academic or sports contexts. These factors can amplify parents’ focus on specific goals and expectations for their children to secure educational prospects. Nonetheless, in contexts where children perceive a secure attachment, interactions with parents tend to foster a supportive and encouraging environment that promotes skill development and mastery success. This finding aligns with previous Western literature (e.g., [65,69,70,71]) exploring the associations between perceived achievement goals or motivational climates and attachment styles in achievement domains such as sports and physical activities. Specifically, most studies suggest that secure internal working models of attachment encourage positive, mastery-focused exploration without excessive concern about failure. Securely attached individuals trust that attachment figures will be available, accepting, and supportive, regardless of the outcome. Conversely, insecure attachment styles may lead to less adaptive, competence-focused exploration, where fear of failure is prominent. This fear arises from a perception that attachment figures are not consistently available, accepting, or supportive, prompting individuals to avoid situations that might diminish their chances of gaining such support.

The patterns of parent–child interactions observed in contexts with contextually different attachments reveal notable contrasts. In insecurely attached contexts, compared to securely attached ones, children often perceive their parents as using psychological control strategies such as invalidating their feelings and demeaning them. These maladaptive strategies are employed to shape children’s behaviors (e.g., meeting parental achievement standards) or emotional responses, which ultimately undermine their sense of autonomy and competence across achievement contexts. Psychological control, regarded by Western scholars [72,73] as a negative parenting approach, interferes with children’s psychological autonomy. By manipulating children’s thoughts and emotions to align their actions with parental expectations, such control often leads to a range of adverse outcomes, including insecure attachment orientations, thwarted psychological needs, anxiety, depressive symptoms, deviant behavior, and poor academic performance (e.g., [72,74,75,76,77,78,79,80,81]). Previous cross-cultural research has highlighted that Chinese parents generally display more controlling behaviors in their parenting compared to Western parents [82,83]. This may be because Chinese parents often assess their self-worth based on their children’s performance, viewing successful child rearing as a significant responsibility [84]. Interpersonal relationships in Chinese culture also emphasize emotional harmony and control [85]. Consequently, children who have been subjected to high levels of parental control from an early age may grow accustomed to it and perceive it as less severe. However, the findings of this study challenge this perspective, suggesting that parental control in these contexts may still have significant negative implications.

This study further revealed that in contexts of insecure attachment, both the behavioral and psychological characteristics of parent–child interactions suggest that parents often display conditional regard toward specific behaviors of their children. However, such maladaptive parenting practices were not observed in contexts of secure attachment, a finding consistent with our initial expectations. Specifically, in insecure attachment contexts, inappropriate parental expectations appear to drive parents to manipulate their children’s self-perception (e.g., feelings of being ignored, undervalued, or unloved) through conditional regard to achieve compliance with their demands. Specifically, this study found that when children fail to meet their parents’ expectations, parents may respond by withdrawing love—through rejection, indifference, or disapproval—toward their child’s need for love. Such responses not only foster maladaptive psychological outcomes in children (e.g., compulsive over-investment and contingent self-worth) but also perpetuate cycles of insecure child–parent attachment. Additionally, these dynamics frustrate the child’s sense of relatedness with their parents, as well as their feelings of autonomy and competence in these contexts.

Our findings align with previous research (e.g., [23,34,39,40,42,43]) exploring the effects of PCPR and PCNR on children’s psychological and behavioral outcomes in achievement contexts. More specifically, the majority of studies reveal that the two parenting practices, PCPR and PCNR, significantly influence children’s and adolescents’ test anxiety, test performance, contingent self-esteem, ability self-concept, psychological need satisfaction, and depressive symptoms. Nevertheless, this study did not observe PCPR as a maladaptive parenting practice across contexts. One potential explanation for this finding is the influence of Confucian values, particularly the emphasis on filial piety, within Taiwanese cultural contexts [86,87]. Filial piety is defined as “providing both emotional and material support to parents… such as respect, love, and attending to their needs, as well as deference and compliance to their wishes…” [46] (p. 317). Consequently, Taiwanese children may perceive the affection, recognition, and attention received from parents—when they meet parental expectations—as a fulfillment of filial piety rather than a strategy of psychological control, as is often suggested in the Western literature. This cultural lens may explain why PCPR, commonly considered a maladaptive parenting practice in Western contexts, is not explicitly perceived as such by Taiwanese children and thus has a different impact on their psychological outcomes. To date, only a limited number of Western studies (e.g., [41]) have examined the relationship between parental conditional regard and attachment dynamics. Moreover, none, if any, studies have explored how PCPR and PCNR influence child–parent attachment relationships and children’s psychological outcomes in Chinese cultural contexts. Future research should delve deeper into these associations to expand our understanding of these dynamics.

## 5. Limitations and Recommendations

This study has made significant efforts to consider potential factors that might influence the findings during the selection of youth participants. These factors include variations in gender, age, type of sport, the gender of the attachment figure, and whether parents had previously served as their child’s sports coach, aiming to enhance the study’s potential as an information-rich source. However, certain factors, such as regional characteristics and parents’ socioeconomic status, were not accounted for and may limit the generalizability of the findings to the broader context of Taiwanese society

Based on our findings, we provide recommendations for parents and practitioners to promote effective parenting practices and support children’s psychological well-being. First of all, parents should not judge their children’s academic abilities solely based on their past academic achievements. This way, parents are less likely to fall into unrealistic expectations and set inflexible and overly high achievement standards for their children. Instead, parents should take children’s learning ability, what specific challenges they meet in their studies, and their academic interests all into consideration so that they can make need-supportive parenting adjustments appropriately and accordingly, such as by being willing to appreciate children’s difficulties in their studies and provide timely and pressure-free assistance in response to their study-related needs. These handy parenting strategies are likely to be conducive to children’s autonomy and confidence in their learning.

Furthermore, parents’ perceived value of children’s participation in sports could bring about different parenting strategies that might contribute to children’s psychological outcomes in their sport. We suggest that parents should put children’s sports interest, willingness, and enjoyment as a priority as this approach helps parents create a task-oriented motivation climate, which is conducive to enhancing children’s autonomy and competence in their sports participation. Once parents focus on the value of utility in sports participation (i.e., how this sport can benefit their children’s future academic career), they may become over-concerned about their children’s success in sports, and that might give rise to their ambition and maladapted parenting behaviors (e.g., manipulativeness, ignorance of their children’s feelings and needs, and being over-responsive toward their performance). This also leads to children no longer simply enjoying the pleasure brought by sports but needing to be concerned about their parents’ emotional and behavioral reactions to the success or failure of their sports performance. While children are busy dealing with their parents’ and their own emotions, it also frustrates their sports competence and autonomy.

## Figures and Tables

**Table 1:** Means and standard deviations of cluster centers for variables included in each cluster analysis.

Variables/Clusters in Each of the Two Contexts	Total Sample M (SD)	Cluster 1 M (SD)	Cluster 2 M (SD)
In the context of sports (cluster analysis 1)	(N = 256)	(n = 121)	(n = 135)
Sporting Security	3.97 (0.75)	3.46 (0.66)	4.43 (0.48)
Sporting Insecurity	2.08 (0.83)	2.73(0.65)	1.49 (0.45)
In the context of academics (cluster analysis 2)	(N = 256)	(n = 129)	(n = 127)
Academic security	3.91 (0.74)	4.35 (0.51)	3.46 (0.66)
Academic insecurity	2.23 (0.78)	1.66 (0.47)	2.80 (0.60)

Note. M (SD): mean (standard deviation).

**Table 2:** Four combination profiles grouped according to cluster analyses of CAS-S and CAS-A attachment ratings.

Group Label	N = 256 (%)	In the Context of Sports	In the Context of Academics
(1) Contextually consistent attachment security	n = 106 (41%)	Cluster 2	Cluster 1
(2) Contextually consistent attachment insecurity	n = 76 (30%)	Cluster 1	Cluster 2
(3) Contextually different attachment (security in sports and insecurity in academics)	n = 51 (20%)	Cluster 2	Cluster 2
(4) Contextually different attachment (insecurity in sports and security in academics)	n = 23 (9%)	Cluster 1	Cluster 1

**Table 3:** Details of case study of youth participants and their parents (n = 8).

Pseudonym	Gender	Age	Child’s Sport	Attachment Figure	Figure Is Coach	Personal Prize	Attachment Characteristic
SS1	M	17	Powerlifting	Mother	No	No	Security in sports/ security in academics
SS2	F	17	Woodball	Father	No	No	
II3	M	10	Triathlon	Mother	No	No	Insecurity in sports/ insecurity in academics
II4	M	12	Triathlon	Mother	No	Yes	
SI5	M	14	Pole Vault	Mother	No	No	Security in sports/ insecurity in academics
SI6	F	11	Triathlon	Mother	No	No	
SI7	M	14	Basketball	Father	Yes	Yes	
IS8	M	14	Table Tennis	Father	Yes	No	Insecurity in sports/security in academics

**Table 4:** Examples of interview guides with children/parents.

1. Do you feel your parent/child behave differently in your sport and academic-related life? Why do you think so?
2. What do your parent/child normally behave in your sport-related activities? (e.g., when talking about sporting-related issues—sports practice sessions, time before games, during games, after games, or any other sport-related interactions)
3. What do your parent/child normally behave in your academic-related activities? (e.g., tutoring courses, homework, and academic-related contests/events.)
4. How do your parent/child normally behave to your sport or academic-related performance/achievement (e.g., good score in exams, win a game, or bad performance in a competition)? Can you make some examples? (e.g., supportive, pressured or neglect) Why?
5. How do your parent/child normally behave in response to your feelings and needs, especially when you feel sad, upset or angry? Can you make some examples relating to your sport or academics? How’s your feeling?
6. Have your parent/child ever done anything that made you feel secure and close to her/him in your sport or academics? What was that? How’s your feeling?
7. Have your parent/child ever done anything that made you feel relaxed knowing that he or she is always there you when engaging sport or academic-related activities? What was that? How’s your feeling?
8. Have your parent/child ever done anything that made you feel like she or him cares about your sport and school life? What was that? How’s your feeling?
9. Have your parent/child ever done anything that made you feel hard to talk about your feelings relating to your sport or academics over with she or him? What was that? How’s your feeling?
10. Have your parent ever done anything that made you have mixed feelings about being emotional close to him or her when doing your schoolwork? What was that? How’s your feeling?

Note. These example questions were a loose guide that served to facilitate in-depth conversations with children/parents. The interviews with parents were also guided by these questions but with slightly adjusted wordings.

**Table 5:** Salient quotations across the contexts within the theme of timely and sensitive responsiveness.

Secure Attachment in the Context of Sports	Secure Attachment in the Context of Academics
(1) When we talk about something relating to my sport, I can always speak freely…I don’t need to worry what I should say and shouldn’t say…it’s pretty free. He always commented on what I said, he didn’t brush me off…I feel relaxed talking with him. (SI7, boy–father bond) (2) Dad and me, we are like good friends…when I come back from my training, I always like to share what happens in my sport with him. (SS2, girl–father bond) (3) She normally talked about her sport training and competition when she came back home…whatever she won or lost, I always told her not to push herself too hard, just do her best…I wouldn’t respond badly if she didn’t do well, it’s just an exercise. (SS2′s father) (4) He didn’t really interfere with what I was doing in my sport, I just need to behave well and don’t do something bad, that’s all…but when I needed his help, he would always try to give it. (SS2, girl–father bond) (5) I didn’t really ask for something in her sport, it all depended on her interests, I was all fine with her choices… I didn’t want to interfere at all …but if she asked something for her sport, I would always support her. (SS2′s father)	(6) I always like to tell her what I have learnt recently, share my learnt knowledge or my school life with her…she always responds to my feelings in a positive and encouraging way…it makes me feel good. (SSI, boy–mother bond) (7) I feel like she cared about my studies, because when I talk my school-related things over with her, she always responds to my feelings, and I think it’s enough for me. I am satisfied she can support what I am doing. (SSI, boy–mother bond) (8) Actually…he didn’t really interfere with my studies, he thought I can handle it well…so he wouldn’t really need to interfere too much…he knew I stayed quite late if I had an exam next day, then he would tell me not to stay up too late…otherwise, he wouldn’t interfere my studies, I could feel his care in this way. (SS2, girl–father bond) (9) My mum’s workload is very heavy, …she didn’t really have time to attend my school-related events, but she cared about them. Like… she would ask about my feelings or how’s it going in school after my exams. (SS1, boy–mother bond) (10) She knew I didn’t do well in my studies, she didn’t really ask for something…but I knew she would always support me if only I didn’t do anything bad. (SS1, boy–mother bond)

**Table 6:** Salient quotations across the contexts within the theme of empathetic concerns.

Secure Attachment in the Context of Sports	Secure Attachment in the Context of Academics
(1) He knew I did training till quite late every day, he asked me to stop at the beginning…but he started supporting me after I told him this is what I want to do and I insisted on it…I think if I hadn’t insisted to do sport, he would have told me to quit. (SS2, girl–father bond) (2) Basically, she would let me decide what I wanted to do and how to do in sport. It’s just she wouldn’t like to see me suffering from my training because she knew our training was quite tough, so she wasn’t really being supportive at the beginning. …but I told her that I like this sport, and I am willing to put effort into it. Yeah…she agreed with me, so she was willing to support my sport. (SS1, boy–mother bond)	(3) I don’t feel he puts pressure on my studies because he knows I am not interested in studying. My friends’ parents always ask for a lot in their studies, like, keep asking them to attend some after-school courses, but my dad doesn’t ask me to do that. (IS8, boy–father bond) (4) I felt he wasn’t interested in studying, he felt doing sport was more interesting…he did poorly in every exam…I told him just focus on the teachers’ tips, just need to pass exams, that’s fine…if teachers aren’t available to deal with your questions, maybe you could ask your coaches or senior teammates during breaks? Just make use of your time to figure it out. It was all I could do to encourage him. (IS8′s father)

## Data Availability

The raw data supporting the conclusions of this article will be made available by the authors upon request.
